# Ocular Gene Therapy: A Literature Review With Focus on Current Clinical Trials

**DOI:** 10.7759/cureus.29533

**Published:** 2022-09-24

**Authors:** Vaibhavi B Wasnik, Archana R Thool

**Affiliations:** 1 Opthalmology, Jawaharlal Nehru Medical College, Datta Meghe Institute of Medical Sciences University (DMIMSU), Wardha, IND; 2 Ophthalmology, Jawaharlal Nehru Medical College, Datta Meghe Institute of Medical Sciences University (DMIMSU), Wardha, IND

**Keywords:** age-related macular degeneration, retinitis pigmentosa, ocular inflammation, viral vectors, ocular gene therapy

## Abstract

Gene therapy has been one of the most researched topics in the last decade. It has now become a revolutionized therapeutic tool of modern medicine. Gene therapy is the alteration of the defective gene involved in the disease process in the host cells. It delivers therapeutic genetic information via modified viral or non-viral vectors. Ocular gene therapy, in particular, has progressed in treating inherited retinal diseases since the eye is a favourable organ for gene therapy development. The advantage of the eye as a target for gene therapy is attributed to its easy accessibility and blood-ocular barrier. Several ongoing clinical trials are investigating various gene therapies for other ocular diseases, including neovascular age-related macular degeneration, retinitis pigmentosa (RP), Usher syndrome, glaucoma, and several others. However, there are challenges such as ocular inflammation and humoral response, infection by the viral vectors, and insertional mutagenesis. These limitations depend on several factors; whether viral or non-viral vectors are used, which viral vectors were used, the route of administration, whether subretinal, intravitreal, or suprachoroidal, and the dose of vectors and the target tissue. These complications may lead to therapeutic failure and vision loss due to intraocular inflammation. This review aims to summarize existing knowledge about ocular gene therapy and the associated limitations we face, with a special focus on a few ongoing clinical trials.

## Introduction and background

Gene therapy is a novel therapeutic approach to managing various inherited and acquired diseases. Gene therapy has already successfully managed (i) inherited diseases such as Leber's congenital amaurosis (LCA), X-linked severe immunodeficiency disease, beta thalassemia, hemophilia, and chronic granulomatous disease; and (ii) acquired diseases such as multiple myeloma, B-cell lymphoma, advanced melanoma, prostate cancer, and many others [1]. The eye is an ideal organ for gene therapy. This is attributed to the fact that it is small, easily accessible, and isolated; it has a blood-retinal barrier; and the other eye can act as a control. It requires a lower dose of vectors. There is little to no chance of systemic infection using viral vectors [2]. Gene therapy can be either ex vivo or in vivo. Ex-vivo gene therapy is when the host cells are collected, cultured, genetically modified, and transplanted back into the host. In vivo means when the genetically modified information is transferred to targeted host cells via viral or non-viral vectors [3]. The viral vectors use the inherent property of viruses to infect the host cell's genomes. The pathological genetic sequence is replaced by the therapeutic genes, which can produce the desired therapeutic effect.

The non-viral vectors transfer either DNA plasmids or small DNA and RNA molecules by physical or chemical methods. Physical methods include electroporation, sonoporation, hydroboration, needles, and DNA ballistics. The chemical methods include using vectors like inorganic particles, lipids, polymers or peptide particles [4]. Ocular gene therapies can be used for various inherited retinal diseases like Leber’s congenital amaurosis, X-linked retinitis pigmentosa (RP), choroideremia, X-linked retinoschisis, Stargardt disease, and Usher syndrome, which are discussed in this review [3]. Various clinical trials for corneal gene therapy are also being done for corneal dystrophies, herpes simplex virus keratitis, Sjogren syndrome, and others [5]. Ocular gene therapy is not only used for inherited diseases but also acquired diseases like glaucoma. Few clinical trials have been conducted for glaucoma where the therapeutic gene, i.e., siRNA, antagonizes β adrenergic receptor synthesis to lower the intraocular pressure [6]. Even if much progress has been made in ocular gene therapy, there are several challenges that we have yet to overcome. This includes uncertainty about the longevity and irreversibility of the therapy. Other challenges include gene therapy complications like ocular inflammation, insertional oncogenesis, or therapeutic failure.

## Review

Gene therapy mechanisms

Gene Replacement/Gene Augmentation

Gene augmentation is most commonly used for autosomal recessive disorders. In these disorders, a defect or absence of a single copy of the gene leads to loss-of-function mutation and thus to an inadequate amount of protein synthesis. In gene augmentation, the abnormal copy of the gene is replaced by the normal copy of the gene via therapeutic vectors. This therapeutic gene can be transferred either as mRNA or as a DNA copy [7]. DNA needs to be injected directly into the nucleus of the cells. It also increases sustained production of the protein, hence it is preferred. The complications of using mRNA include instability of the mRNA molecule due to changes in sequence within it and the induction of immune responses. The disadvantage of gene augmentation is that it cannot be used in an already degenerated retina. This technique has been successfully used for Food and Drug Administration (FDA) approved Phase 3 trial of Luxturna, a gene product used to treat Leber's congenital amaurosis targeting gene RPE65. The RPE65 gene encodes for retinoid isomerohydrolase, an enzyme of the visual cycle synthesized by the retinal pigment epithelium (RPE) [7,8].

Gene Silencing/Gene Editing

This mechanism is used for autosomal dominant inherited diseases. Here, the mutation is a gain-of-function mutation. The disease occurs due to the expression of undesired proteins or gene products of the mutated gene. The aim is to prevent the mutated gene from expressing and encoding the undesired protein. This can either be allele-specific or non-specific. In allele-specific, only the mutated allele is targeted. In allele non-specific, both the mutated and the functional allele are silenced and, by gene augmentation, replaced with the normal gene. It can be done at three levels of the genetic machinery: (1) DNA, (2) RNA, and (3) transcription [3].

DNA-based genome editing techniques

CRISPR/Cas9

CRISPR are clustered regularly interspaced short palindromic repeats of prokaryotic DNA. The virus genome follows each repetitive sequence from a previous infection, known as spacer DNA. Cas9 is a CRISPR-associated protein 9 that specifically cuts DNA at these recognizing sites, leading to gene silencing [9]. When used for genome editing, Cas9 endonuclease, along with guide RNA, is injected into the nucleus of target cells. The RNA-guided endonuclease cuts the double-stranded DNA at targeted sites, activating the DNA repair system [3]. This technique has been used therapeutically for autosomal dominant RP [10].

Other DNA-based genome editing techniques are transcription activator-like effector nucleases (TALENs) and zinc finger nucleases (ZFN) [11]. There are ongoing clinical trials to treat acquired immune deficiency syndrome using the ZFN genome editing technique targeting the C-C chemokine receptor type 5 gene (CCR5 gene) of CD4+T cells (T helper cells). *CCR5* is present on the surface of these cells and acts as a coreceptor for the human immunodeficiency virus. In this trial, the *CCR5* gene was silenced by genome editing using the ZFN technique [12].

RNA-based genome editing techniques 

These techniques work either by eliminating mRNA molecules or preventing their translation.

Small Interfering RNA

RNA interference (RNAi) is a post-transcriptional gene silencing technique that uses sequence-specific siRNA to cleave targeted RNA. The RNAi pathway starts with long pieces of dsRNA being cleaved into small interfering RNA by the endoribonuclease dicer enzyme. This step can be skipped by directly administering siRNA into the targeted cell cytoplasm. Once in the cytoplasm, the siRNA gets incorporated into a protein complex called the RNA-induced silencing complex (RISC). The double-stranded siRNA gets cleaved into sense and antisense strands. The antisense strand guides the RISC to the targeted mRNA and cleaves it, preventing gene expression. RNA interference is currently under trial for managing age-related macular degeneration, glaucoma, RP, and diabetic retinopathy [3,13].

Antisense Oligonucleotide

These are complementary strands of the targeted mRNA molecule. It causes the downregulation of gene expression by two mechanisms. An antisense oligonucleotide binds to the targeted mRNA and forms a complex. The mRNA and antisense oligonucleotide complex are cleaved by RNaseH1 activity. The other mechanism acts by translation inhibition, preventing exon splicing, 5'mRNA capping, or destabilizing the RNA. Currently, the antisense oligonucleotide is under trial for ocular gene therapy for Leber’s Congenital Amaurosis acting on the gene CEP290. This gene encodes for centrosomal protein 290 [14,15].

Viral vector 

Characteristics of the vectors are summarized in Table 1.

**Table 1:** Characteristics of viral vectors AV: adenovirus, AAV: adeno-associated virus, LV: lentivirus Modified from references [18,19]

	AV	AAV	LV
Type of virus	dsDNA virus	ssDNA virus	ssRNA virus
Packaging of genetic material	8kb	<5 kb	8 kb
Immunogenicity	Highly immunogenic	Mildly immunogenic	Moderately immunogenic
Insertional mutagenesis	Possible	Less likely as it delivers materials in extra-genomic circular episome	Most likely as it integrates cDNA (complementary DNA) into the chromosome of the target cell.
Advantages	Transduction of most tissues, especially retina and anterior segment, ease of genetic manipulation	Not immunogenic not pathogenic stable long-term gene expression	With sustained gene expression, large-sized therapeutic genes can be packed.
Disadvantages	Strong inflammatory responses, pathogenic, cause systemic infection	It cannot carry large-sized genetic material	Induce oncogenesis
Used in	Retinoblastoma [16]	Most the clinical trials age-related macular degeneration, retinitis pigmentosa	Stargardt disease Usher syndrome [17]

Ocular gene therapy: current clinical trials

Several ongoing clinical trials are conducted by ocular gene therapy ranging from retinal to corneal diseases [20]. Table 2 summarizes a few clinical trials, including completed and ongoing trials, which are discussed further.

**Table 2:** Ongoing clinical trials for ocular gene therapy VEGFA: vascular endothelial growth factor-A, AMD: age-related macular degeneration, DR: diabetic retinopathy, rAAV: recombinant adeno associated virus, RP: retinitis pigmentosa, IDLV: integration-deficient lentiviral vector [20]

Disease	Iintervention	Participants	Route	Clinical trial ID
Neovascular age-related macular degeneration diabetic macular edema retinal vein occlusion	Genetic: BD311 IDLV expressing VEGFA antibody	018	Suprachoroidal	NCT05099094
Choroideremia	Biological: injection of AAV2-REP1 (10e11 VG)	006	Subretinal	NCT02553135
Choroideremia	Drug: BIIB111	066	Subretinal	NCT03507686
Choroideremia	Genetic: rAAV2.REP1 vector	006	Subretinal	NCT02077361
Neovascular AMD	Biological: RGX-314	095	Suprachoroidal	NCT04514653
DR	Genetic: RGX-314 dose	060	Suprachoroidal	NCT04567550
Choroideremia	Genetic: BIIB111	170	Sub-retinal	NCT03496012
Achromatopsia	Biological: adeno-associated virus vector AAV- CNGA3	011	Sub-retinal	NCT03758404
Herpetic viral keratitis	BD111 CRISPR/Cas9 mRNA	006	Corneal	NCT04560790
Bietti's crystalline dystrophy	Drug: gene replacement therapy by rAAV2/8-hCYP4V2	003	Sub-retinal	NCT04722107
Diabetic macular edema diabetic retinopathy	Gene product: 6E11 VG/eye of ADVM-022 gene product: 2E11 VG/eye of ADVM-022	036	Intravitreal	NCT04418427
Retinitis pigmentosa	Gene therapy product-vMCO-010 (optogenetic therapy)	027	Intravitreal	NCT04945772
Non-syndromic retinitis pigmentosa	Combination product: gene therapy: GS030-DP medical device: GS030-MD (optogenetic therapy)	015	Intravitreal	NCT03326336
Wet age-related macular degeneration neovascular age-related macular degeneration	Gene product: ADVM-022	030	Intravitreal	NCT03748784
X-linked retinoschisis	Biological: RS1 AAV vector	012	Intravitreal	NCT02317887
MERTK-associated (RP)	rAAV2-VMD2-hMERTK recombinant adeno-associated virus	006	Subretinal	NCT01482195
Autosomal dominant retinitis pigmentosa	Drug: QR-1123	011	Intravitreal	NCT04123626
X-linked retinoschisis	Biological: rAAV2tYF-CB-hRS1	027	Intravitreal	NCT02416622
Leber congenital amaurosis	Genetic: rAAV2-hRPE65	003	Subretinal	NCT00821340
Recurrent retinoblastoma	Oncolytic adenovirus VCN-01	013	Intravitreal	NCT03284268
X-linked retinitis pigmentosa (RPGR gene)	AAV 4D-125	043	Intravitreal	NCT04517149

Leber's congenital amaurosis

LCA is a childhood-onset autosomal recessive disease that leads to vision loss. It occurs due to the mutation of several genes, especially RPE65, encoding for retinoid isomerohydrolase, which is predominantly expressed in the retinal pigmented epithelium. This mutation leads to a deficiency of the enzyme retinoid isomerase, which is responsible for chromophore formation. Chromophore forms visual pigments in photoreceptors of the retina. This leads to visual impairment [18].

The first approved ocular gene therapy by the FDA is the phase 3 trial of Luxturna sub-retinal injection for LCA in both eyes. The gene product was AAV2-hRPE65v2 (voretigene neparvovec-rzyl), and the vector was adeno-associated virus 2 (NCT00999609) [21]. The inclusion criteria consisted of participants being three years or older, being a diagnosed case of LCA with RPE65 mutation, visual acuity of less than 20/60 in both eyes with the best possible correction. The subjects should be evaluated by multi-luminance mobility testing (MLMT). The most important inclusion criteria were the presence of viable retinal cells as determined by optical coherence tomography (OCT). The outcome was measured by MLMT, which is used to measure functional vision changes. The MLMT score ranged from 0 to 6, with six being where the subject was able to pass MLMT with low light intensity. The MLMT score change was a difference between the baseline score and the score measured after a year. The other measures for the trial's outcome were full-field light sensitivity threshold testing and visual acuity. The trial results indicated that the MLMT change score in the interventional group was 1.8 as opposed to the control group, with an MLMT change score of 0.2. This indicated an improvement in functional vision by Voretiegene Neparvovec gene replacement therapy [8]. The adverse effect has been described in Table 3.

**Table 3:** Adverse effects of voretiegene neparvovec gene replacement therapy [8]

Adverse effects	Participants (n=20)	Incidence
Ocular inflammation	2/20	6
Cataract	3/20	4
Increased intraocular pressure	4/20	5
Retinal tear	2/20	2
Adverse drug reaction	2/20	2
Convulsions	1/20	1

The only serious complications were convulsions in one participant out of 20 participants with a history of pre-existing seizure disorder and adverse drug reactions in a participant with a history of complicated oral surgery and pre-existing seizure disorder. The gain in visual function has been present for over three years. However, the durability of the intervention is still not determined [8]. Other clinical trials for LCA are mentioned in Table 4.

**Table 4:** Ongoing clinical trials for Lebers congenital amaurosis *RPE65*: encodes protein retinoid isomerohydrolase, *CEP290*:encodes centrosomal protein 290 [20]

Clinical trial ID	Gene involved	Intervention	Participants	Route
NCT00999609	REP65	Biological: AAV2-hRPE65v2 voretigene neparvovec-rzyl	31	Subretinal
NCT03140969	CEP290	QR-110 RNA antisense oligonucleotide	11	Intravitreal
NCT00749957	REP65	Biological: rAAV2-CB-hRPE65	12	Subretinal
NCT00643747	REP65	Biological: tgAAG76 (rAAV2/2.hRPE65p.hRPE65)	12	Subretinal
NCT03872479	CEP290	EDIT-101	34	Subretinal

Retinitis pigmentosa

RP is a group of disorders that cause progressive retinal dystrophy and vision loss. It is one of the most common causes of vision loss. One in every 4000 individuals worldwide is affected by RP. RP can be either autosomal dominant, autosomal recessive, or X-linked. Autosomal recessive is the most common. More than 70 genes are found to be concerned with the development of retinitis pigmentosa [18].

Optogenetic Therapy for Advanced Retinitis Pigmentosa

The clinical trial (NCT03326336) is the most advanced novel ongoing clinical trial for the management of advanced stages of retinitis pigmentosa, combining gene therapy, engineering, and mechanics. It does not target mutated photoreceptors; thus, the mutation is independent. It targets retinal ganglion cells, bypassing the rest of the pathway. The ganglion cells are injected with an optogenetic vector, AAV2.7m8, which encodes for light-sensing proteins CrimsonR and tdTomato. This transgene vector is injected via a single intravitreal injection followed by the use of engineered goggles, GS030MD, that sense light changes in their vicinity and project them into the genetically modified ganglion cells. The objective of this clinical trial was to check the safety of the gene product and the recovery of vision. The clinical trial till now has reported partial vision recovery in one out of 15 participants. The participant, using light-stimulating goggles following gene therapy, could perceive and locate the objects. The therapy was well tolerated, and no intraocular inflammation or other changes were noted. This indicates that optogenetic gene therapy, along with light-stimulating goggles, can be used to partially restore vision in advanced RP [22,23].

Autosomal Dominant Retinitis Pigmentosa

RHO (Rhodopsin) gene was responsible for about 25% of all cases of autosomal dominant retinitis pigmentosa (ADRP). The RHO gene transcribes the RHO protein. RHO protein is the visual pigment present in the rods' outer segments. There are two possible clinical scenarios in ADRP. In class A, there is an early presentation with severe progressive loss of rods. The main aim of the therapy here is to preserve the functions of the cones. In class B, there is a slow progression, and the functions of the rods are well preserved. Here, the main aim of the therapy will be the preservation of the rods. The most common mutation in the RHO gene is the substitution from proline to histidine at the 23rd position (P23H) [10]. The ongoing clinical trial, NCT04123626, targets the P23H mutation of the RHO gene. The gene product used here is QR-1123. It is an antisense oligonucleotide, which is an ssDNA molecule complementary to the targeted P23H mRNA, which increases expression of the wild type of the RHO protein in photoreceptors [24].

MERTK-Associated Retinitis Pigmentosa

One of the other genes involved in the pathogenesis of RP is the MER tyrosine kinase gene (MERTK). The photoreceptors in the retinal epithelium recycle their outer segments. The shed outer segments of the photoreceptors are phagocytized by MERTK [25]. The current clinical trial for MERTK-associated RP (NCT01482195) delivers unilateral subretinal rAAV encoding MERTK protein. This trial aimed to assess the safety of the gene product rAaV2-VMD2-hMERTK. The collected data indicated that three out of six participants showed visual improvement, which lasted for two years in two of these participants. The adverse effects of this clinical trial are summarized in Table 5. Other than these adverse effects, the clinical trial was found to be safe.

**Table 5:** Adverse effects of gene product rAaV2-VMD2-hMERTK [26]

Adverse effects	Participants involved	Incidence
Filamentary keratitis	1/6	1
Progressive cataract, oscillopsia	1/6	1
AAV antibodies	1/6	1
No adverse effects	3/6	-

Age-related macular degeneration

Age-related macular degeneration is one of the most common causes of irreversible blindness. It consists of two phases: (1) dry or non-neovascular and (2) wet or neovascular. During the non-neovascular phase, there is atrophy of the retinal cells in patches, called geographic atrophy, leading to loss of central vision. During the neovascular phase, there is the formation of new blood vessels originating from the choroid into the sub-retinal space. These vessels cause leakage of fluid into the subretinal space as they lack tight junctions. The subretinal space is filled with fluid, leading to oedema and reversible vision loss. However, if this fluid build-up continues for several months, it may cause irreversible vision loss [27]. The vascular endothelial growth factor is responsible for the proliferation of new vessels. Hence, the current standard therapy for neovascular macular degeneration is intravitreal drug administration of anti-VEGF agents. However, considering the economic and social burden this therapy puts on because of the repeated intravitreal injections, complications, high drug cost, and repeated imaging, ocular gene therapy for sustained drug delivery have become necessary [28].

The current clinical trials can have two mechanisms: either by the sustained release of anti-angiogenic factors or by gene silencing for factors that cause overexpression of VEGF [29]. The current trials are summarized in Table 6.

**Table 6:** Clinical trials for age-related macular degeneration [20]

Clinical trial ID	Gene involved	Intervention	Participants	Route	Vector
NCT00109499	PDF	AdGVPEDF.11D	28	Intravitreal	Adenovirus
NCT03585556	sCD59	Biological: AAVCAGsCD5 Drug: anti-VEGF drug: oral prednisolone	25	Intravitreal	AAV2
NCT01494805	sFLT01	rAAV.sFlt-1 (dose escalating)	40	Subretinal	AAV2
NCT01301443	Endostatin and angiostatin	RetinoStat	21	Subretinal	Lentivirus
NCT01024998	sFLT01	AAV2-sFLT01	19	Intravitreal	AAV2
NCT03748784	Aflibercept	ADVM-022	30	Intravitreal	AAV2
NCT03066258	Anti-VEGF Fab	RGX-314 (dose escalation)	42	Subretinal	AAV8

Pigment Epithelium-Derived Factor

The first clinical trial for neovascular age-related macular degeneration was conducted by targeting the gene for pigment epithelium-derived factor (PEDF) protein. The NCT number for the clinical trial is NCT00109499 [30]. PEDF is usually present in the eye, acting as an anti-angiogenesis factor. Its levels are altered in neovascular macular degeneration. The transgene was delivered via the intravitreal route as AAV2 expressing PEGF. The adverse effects occurred in 25% of the cohort population and were limited to mild ocular inflammation and a slight increase in intraocular pressure, which were easily manageable. The results of this trial had a dose-related effect. The cohorts receiving the dose of 108 particle units showed no increase in the lesion and a significant decrease in neovascularization. Whereas the cohorts receiving doses less than 108 showed an increase in the size of lesions by one disk area at 12 months. This suggests that this ocular gene transfer is a feasible approach and further studies should be carried out [31].

Aflibercept

Aflibercept is an anti-VEGF factor that acts as a receptor for VEGF-A, VEGF-B, and placental growth factors, preventing neovascularization. Aflibercept is a fusion protein encoded by different genes [29]. The clinical trial (NCT03748784) studied the safety and efficacy of the gene product ADVM-022, responsible for the sustained release of aflibercept. The vector used here is AAV-2, administered by the intravitreous route. After 34 weeks, ADVM-022 was well tolerated. Only mild ocular inflammation was observed and resolved by steroid eye drops. Consistent improvements were seen on OCT, and patients maintained vision throughout [32].

Endostatin and Angiostatin

Endostatin and angiostatin inhibit angiogenesis endogenously. The clinical trial (NCT01301443) is a dose escalation study to determine the safety and efficacy of a lentiviral vector administered subretinally for sustained expression of endostatin and angiostatin [33]. The procedure caused a macular hole in one of the participants. However, it was very well tolerated by others. Eight participants showed sustained expression of angiostatin and endostatin for a period of 2.5 years, whereas two participants showed sustained release for four years [29,34].

sFLT-1

FLT-1 is a receptor gene that endogenously inhibits VEGF-A, preventing angiogenesis. Currently, there are two clinical trials that focus on viral vector-delivered sFLT-1.

The clinical trial (NCT01494805) delivers rAAV.sFlt-1 via the AAV2 vector subretinally, which encodes naturally occurring FLT-1. No particular adverse effects were seen. It was found to be safe and tolerable, especially among the geriatric population, and could help decrease the frequency of anti-VEGF injections. However, no significant improvement in visual acuity or other exploratory points was observed [28,35].

The other clinical trial (NCT01024998) delivered AAV2-sFLT01 via the vector AAV-2 by the intravitreous route. The viral vector encoded a fusion protein of sFLT-1 domain two and the Fc domain of Immunoglobulin G1. It was a dose escalation study, which showed that the gene product was well tolerated at all doses. It was observed that 5 out of 10 participants who were administered higher doses showed a detectable amount of sFLT-1 levels. The participants, who did not express sFLT-1, had an antibody titre of 1:400 against the AAV-2 vector. The clinical trial did not particularly show any improvement in visual acuity or retinal thickness [28,29,36].

Immune respone

One of the major setbacks of ocular gene therapy is inflammation. The eye is considered a site of immune privilege due to varying factors like the retinal-blood barrier. However, the immunogenicity of viral vectors, their capsids, the transgene, and the transgene product as a foreign body can activate immune responses. Various factors affect the severity of the immune response, such as viral vectors used, administration route, and viral dose, including several others [37].

*Type of V* *ector*

AV is currently only used for the gene therapy of retinoblastoma as it is highly immunogenic. It causes severe inflammation and destruction of the transduced cell. Being a double-stranded virus, it binds strongly to TLR9 and activates a stronger immune response.

AAV has several serotypes which have different levels of immunogenicity. Most of the population normally has pre-existing antibodies against AAV2, and a small part of the population has antibodies against AAV8. However, cross-reactivity between serotypes is possible. AAVs only generate favourable immune responses, hence they are the preferred vector for ocular gene therapy [37].

LV generates a stronger immune response than an adeno-associated virus but is preferred when more genetic material needs to be transduced [18,38].

Route of Administration

Different routes of delivery expose the vector to different systemic and local biodistribution. The intravitreal route is the most commonly used but shows a higher immune response. This is because the viral particles from the vitreous, through Schlemm’s canal and reach the systemic circulation and lymphatic flow and can activate an immune response. The subretinal space is relatively immune-privileged and shows a very mild immune response [37].

Viral Dose

According to Timmers et al., the relationship between the viral dose and ocular inflammation depends upon the site of inflammation. In the anterior chamber, the dose escalation did not show any effect. However, in the vitreal chamber, dose escalation showed a greater degree of immune response [39].

## Conclusions

The basis of gene therapy is to replace or inactivate a faulty gene or administer a gene product that can prevent the disease process. Ocular gene therapy, in particular, has proven to be a promising tool to treat many inherited retinal diseases like RP, LCA, choroideremia, Stargardt disease, and acquired diseases like nAMD, glaucoma, and diabetic retinopathy. Several clinical trials are being conducted, though in their early stages, have shown promising results. The aim of any clinical trial is to assess the safety and efficacy of the gene product. Something to be concerned about when it comes to the safety of ocular gene therapy is inflammation. There are several factors that affect it such as the vectors used, the route involved, and the dose of the gene product. The subretinal route generates a milder immune response compared to the intravitreal route. AAVs are currently preferred, though newer viral vectors should be considered. As we already know what factors affect it, vectors and delivery methods need to be developed to prevent ocular inflammation. Factors that influence the selection of a vector are immunogenicity, size, the amount of genetic material it can carry and the adverse effects it generates, if any. There are several unanswered questions when it comes to the sustainability of ocular gene therapy. We still do not know how long the effect of gene therapy will last, what factors will affect it and how they can be influenced. Another issue that needs addressing is the long-term adverse effects of gene therapy.

As we have seen in the review, there are several gene targets when it comes to nAMD. A combined approach with several gene targets for such diseases can be developed. A thorough study of the molecular genetics of the disease needs to be done to have a positive outcome in such a case. Though ocular gene therapy has made massive progress in retinal diseases, gene therapy for other ocular diseases like uveitis, corneal graft rejection, and corneal genetic dystrophies needs to be addressed. Ocular gene therapy is an emerging and promising field that can change the trajectory of how ocular diseases will be treated in the future.
